# Editorial: Roles of Sleep Disruption and Circadian Rhythm Alterations on Neurodegeneration and Alzheimer's Disease

**DOI:** 10.3389/fnins.2021.737895

**Published:** 2021-09-06

**Authors:** Marilyn J. Duncan, Sigrid C. Veasey, Phyllis Zee

**Affiliations:** ^1^Department of Neuroscience, University of Kentucky College of Medicine, Lexington, KY, United States; ^2^Chronobiology and Sleep Institute and Department Medicine, Perelman School of Medicine, University of Pennsylvania, Philadelphia, PA, United States; ^3^Department of Neurology, Northwestern University Feinberg School of Medicine, Chicago, IL, United States

**Keywords:** sleep, circadian rhythms, neurodegeneration, Alzheimer's disease, Parkinson's disease, obstructive sleep apnea, traumatic brain injury

Striking changes in circadian rhythms, including the sleep-wake rhythm, characterize many neurodegenerative processes, e.g., Alzheimer's disease (AD), Parkinson's disease, Huntington's disease, and traumatic brain injury (TBI). Because disturbances in circadian rhythm and sleep can precede the overt cognitive impairment, they have been postulated as risk factors or predictors of these diseases (Musiek and Holtzman, 2016).

Circadian rhythms represent biological adaptations to dramatic and predictable daily changes in the physical environment experienced by virtually all organisms living on earth. By definition, circadian rhythms have an intrinsic period length of ~24 h, are endogenously generated (i.e., persist under constant environmental conditions), are temperature compensated (i.e., the period is little affected by temperature) and can be entrained by temporal cues (zeitgebers). Although the light-dark cycle is the most robust zeitgeber, other factors including activity and timing of food can modify circadian phase. Circadian rhythms govern the rhythmic expression of a large portion of the genome (Zhang et al., 2014) and a wide range of cellular, physiological, and behavioral processes, including the daily sleep-wake rhythm.

Sleep is regulated not only by the circadian phase but also by the time elapsed since prior sleep (the homeostatic sleep drive), according to the two-process model of sleep regulation (Borbély, 1982). Although these processes were initially considered to be independent, they have since been shown to influence each other. Sleep deprivation can alter the phase of circadian rhythms (Antle and Mistlberger, 2000) and the expression of circadian clock genes (Franken et al., 2007). Destruction of the suprachiasmatic nucleus (SCN), the master mammalian circadian pacemaker, not only ablates the daily sleep-wake rhythm but also increases the total amount of sleep in squirrel monkeys (Edgar et al., 1993) and non-REM sleep in mice (Easton et al., 2004). Thus, loss of sleep affects the timing of circadian rhythms while circadian rhythm alterations impair sleep, underscoring a complex relationship between these processes (Mistlberger, 2005; Morin, 2013; Scammell et al., 2017).

Neural circuits regulating sleep and circadian rhythm output are vulnerable to brain damage and neurodegeneration (Coogan et al., 2013; Theofilas et al., 2017). For example, accumulation of amyloid-beta disrupts circadian rhythms and sleep (Tate et al., 1992; Roh et al., 2012). On the other hand, disruption of circadian rhythms or sleep deleteriously affects many physiological, behavioral, and neuronal functions (Musiek and Holtzman, 2016; Zhu et al., 2016). Current evidence suggests that the bi-directional interactions between neurodegeneration and disruption of circadian rhythms and sleep exacerbates neuropathology in AD and other neurological disorders (Wang and Holtzman, 2020). In this special issue, discrete circadian rhythm alterations in these disorders and their associations with specific neuropathological processes are described in articles by Fifel and Videnovic and Green et al.

Sleep disruptions appear prior to the prodromal stages of AD, and include fragmentation of the daily sleep-wake rhythm, reduced total sleep, and loss of deep sleep (i.e., slow wave sleep) (Tranah et al., 2011; Guarnieri et al., 2012; Lim et al., 2013; Guarnieri and Sorbi, 2015). Two articles in this issue focus on AD-related changes in discrete neuronal oscillations. As Mander describes, AD neuropathology is accompanied by deficits in three types of local sleep oscillations: (1) frequency-specific frontal slow-wave expression during non-REM sleep, (2) parietal sleep spindles expression, and (3) the quality of electroencephalographic desynchrony in REM sleep. The association of these specific sleep deficits with amyloid-beta and tau pathology and their potential roles in sleep-dependent memory processes and cognitive changes in AD are discussed. The mechanistic importance of AD-associated deficits in slow wave activity is emphasized in the article by Lee et al., which reviews studies showing that the frequency of slow waves affects memory consolidation, amyloid deposition, and neuronal calcium homeostasis. Slow wave sleep is proposed as a therapeutic target for AD.

Variations in neuronal oscillatory activity also occur in patients with moderate obstructive sleep apnea (OSA), a common breathing disorder associated with brain injury and memory impairment. As described by Zhou et al. in this issue, dynamic changes in EEG spectral power occur during respiratory events, including cortical hyperactivation during N2 sleep, suppression of β-band information transmission, abnormal interhemispheric effective connectivity, and intrahemispheric “rise-to-down” fluctuations in the γ band. This work provides insights into how OSA affects cognition and neuropsychiatric function.

To enable preclinical investigations of therapeutic strategies to ameliorate sleep and circadian dysfunction in AD, animal models that temporally replicate these disturbances and neurodegeneration are needed. While some AD mouse models exhibit changes in sleep and circadian rhythms resembling those seen in AD patients, these changes are not consistent across the models, as described by Sheehan and Musiek in this issue. This review provides a fine-grained analysis of the expression of circadian rhythms reported from various models and provides suggestions on how to standardize and optimize future studies of circadian rhythms in AD mouse models. Also in this issue, Lippi et al. describe daily activity rhythms and their responses to zinc in a novel mouse model that exhibits amyloid and tau pathology based on a cross between the J20 (hAPP) and P301L (Tau) lines. Sare et al. report on the TgF344-AD rat model of AD that expresses age-dependent tau and amyloid pathology. Age-related changes in rest-activity rhythms, cognitive performance, and hyposmia are exhibited by the TgF344-AD rats with interesting sex-dependent effects.

Several article in this issue described basic research focused on the underlying mechanisms linking sleep and circadian rhythm dysfunction to neurodegeneration. Sharma et al. review this topic with a special focus on the effect of aberrant circadian rhythmicity on E3 ligase and poly adenosine disphophate (ADP-ribose) polymerase 1 activity in Parkinson's disease. Cassar et al. developed a knock-in Drosophila model of tauopathy and reveal that mutant tau disrupts daily sleep rhythms independent of circadian rhythm changes by interfering with central pacemaker neuron output connectivity. Green et al. describe the role of sleep disruption-associated inflammation in mediating the increased risk of AD occurring with traumatic brain injury. Todd focuses on the potential neural pathways by which circadian rhythm disruption may cause the aggression and agitation that AD patients exhibit during “sundowning syndrome.”

Together, the articles presented in this special issue highlight important questions to address regarding interactions between sleep, circadian rhythms, and neurodegeneration and describe newer models and approaches that may facilitate this work. Overall, the work substantiates the concept that study of neural consequences of circadian and sleep disturbances will provide insight into molecular bases of neurodegenerative processes, while study of sleep and circadian rhythms in animal models and humans with neurodegeneration will provide fundamental knowledge of sleep and circadian neurobiology. These efforts will meet with the greatest success when experts in all three fields collaborate.

## Author Contributions

MD wrote this editorial. SV and PZ provided revisions.

## Conflict of Interest

The authors declare that the research was conducted in the absence of any commercial or financial relationships that could be construed as a potential conflict of interest.

## Publisher's Note

All claims expressed in this article are solely those of the authors and do not necessarily represent those of their affiliated organizations, or those of the publisher, the editors and the reviewers. Any product that may be evaluated in this article, or claim that may be made by its manufacturer, is not guaranteed or endorsed by the publisher.
